# Lake surface cooling drives littoral-pelagic exchange of dissolved gases

**DOI:** 10.1126/sciadv.adi0617

**Published:** 2024-01-24

**Authors:** Tomy Doda, Cintia L. Ramón, Hugo N. Ulloa, Matthias S. Brennwald, Rolf Kipfer, Marie-Elodie Perga, Alfred Wüest, Carsten J. Schubert, Damien Bouffard

**Affiliations:** ^1^ Eawag, Swiss Federal Institute of Aquatic Science and Technology, Department of Surface Waters–Research and Management, Kastanienbaum, Switzerland.; ^2^ Limnology Center, École Polytechnique Fédérale de Lausanne, Lausanne, Switzerland.; ^3^ Department of Civil Engineering, University of Granada, Granada, Spain.; ^4^ Department of Earth and Environmental Science, University of Pennsylvania, Philadelphia, USA.; ^5^ Eawag, Swiss Federal Institute of Aquatic Science and Technology, Department of Water Resources and Drinking Water, Dübendorf, Switzerland.; ^6^ Institute of Biogeochemistry and Pollutant Dynamics, Swiss Federal Institute Technology, ETH Zurich, Zurich, Switzerland.; ^7^ Institute of Geochemistry and Petrology, Swiss Federal Institute Technology, ETH Zurich, Zurich, Switzerland.; ^8^ Faculty of Geoscience and Environment, Institute of Earth Surface Dynamics, University of Lausanne, Lausanne, Switzerland.

## Abstract

The extent of littoral influence on lake gas dynamics remains debated in the aquatic science community due to the lack of direct quantification of lateral gas transport. The prevalent assumption of diffusive horizontal transport in gas budgets fails to explain anomalies observed in pelagic gas concentrations. Here, we demonstrate through high-frequency measurements in a eutrophic lake that daily convective horizontal circulation generates littoral-pelagic advective gas fluxes one order of magnitude larger than typical horizontal fluxes used in gas budgets. These lateral fluxes are sufficient to redistribute gases at the basin-scale and generate concentration anomalies reported in other lakes. Our observations also contrast the hypothesis of pure, nocturnal littoral-to-pelagic exchange by showing that convective circulation transports gases such as oxygen and methane toward both the pelagic and littoral zones during the daytime. This study challenges the traditional pelagic-centered models of aquatic systems by showing that convective circulation represents a fundamental lateral transport mechanism to be integrated into gas budgets.

## Teaser

Cooling-induced horizontal circulation redistributes gases daily between littoral and pelagic lake waters under calm conditions.

## INTRODUCTION

**Fig. 1.**
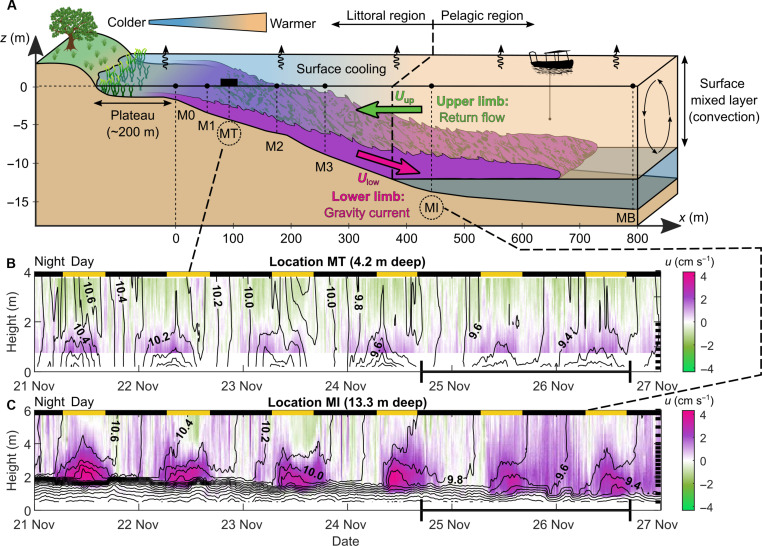
Differential cooling drives diurnal cross-shore convective circulation. (**A**) Schematic of the two-limb circulation along a cross-shore transect. The lower limb (purple) is the downslope gravity current with velocity *U*_low_ that intrudes the base of the mixed layer. The upper limb (green) is the opposite surface flow of velocity *U*_up_ directed toward the shore. The *x* axis starts at the end of the plateau, and the plateau is not to scale in the schematic. Vertical dashed lines indicate the location of the seven measurement stations in Rotsee, where gas concentrations were measured by two portable mass spectrometers (*38*) installed at MT (platform) and deployed from a boat. Circulating arrows in the surface mixed layer depict convection induced by surface cooling. (**B** and **C**) Six-day time series of cross-shore velocity as a function of height above sediment measured in the littoral region (MT, 4.2 m deep) (B) and pelagic region (MI, 13.3 m deep) (C). Note that the *y* axes have a different scale in (B) and (C). Black lines are 0.1°C spaced isotherms from linearly interpolated temperature between each thermistor. Black ticks on the right axis indicate the vertical location of the thermistors. Day and night periods are shown by black and yellow rectangles, respectively. The gas experiment duration from 24 to 26 November 2020 is scaled in bold on the *x* axis.

Lakes provide critical ecosystem services that mostly derive from their littoral zones (*1*, *2*). Traditional models for lake biogeochemistry, however, consist of one-dimensional vertical frameworks that assume lakes functioning from their deep pelagic zone, excluding the littoral zone (*3*, *4*). Although these one-dimensional models can predict long-term trends to a first order, neglecting the effects of lateral boundaries may obscure critical biogeochemical fluxes and their global-scale implications (*5*). A first step toward a more accurate, eventually three-dimensional, representation of biogeochemical fluxes consists in adding the littoral-pelagic dimension to vertical models. Such a two-dimensional approach includes the effects of lateral advection and horizontal turbulent diffusion on dissolved constituents. Lateral advection of dissolved constituents is controlled by cross-shore currents, whereas horizontal turbulent diffusion of such constituents results from localized turbulent eddies. The combination of turbulent motions and the large-scale flow field leads to the irreversible and efficient spreading of dissolved constituents, a process we here denote as horizontal dispersion (*6*). The advective and turbulent diffusive fluxes modulate the temporal dynamics of any dissolved constituent of concentration *C* according to the two-dimensional advection-diffusion-reaction equation (*7*)

$$\frac{\partial C}{\partial t}=R-\frac{{\partial F}_{\text{adv},x}}{\partial x}-\frac{{\partial F}_{\text{adv},z}}{\partial z}-\frac{{\partial F}_{\text{turb}\_\text{diff},x}}{\partial x}-\frac{{\partial F}_{\text{turb}\_\text{diff},z}}{\partial z},$$ (1)

where (*x*, *z*) are the horizontal and vertical coordinates (Fig. 1A); *R* is the sum of all reaction rates; *F*_adv,*x*_ and *F*_adv,*z*_ are the horizontal and vertical advective fluxes, respectively; and *F*_turb_diff,*x*_ and *F*_turb_diff,*z*_ are the horizontal and vertical turbulent diffusive fluxes, respectively. The horizontal turbulent diffusive flux can be modeled as *F*_turb_diff,*x*_ = −*K_x_∂C*/∂*x*, where *K_x_* is the horizontal turbulent diffusion coefficient (also called horizontal dispersion coefficient) that increases with patch size (*6*). In greenhouse gas budgets and ecosystem metabolism estimates, the horizontal advective flux *F*_adv,*x*_ is commonly neglected (*5*, *8*) and lateral transport is only modeled as lateral dispersion by estimating the horizontal turbulent diffusion flux *F*_turb_diff,*x*_ (*9*–*11*). These lateral fluxes estimates fail to generate gas anomalies observed in the pelagic zone, including methane peaks in the oxic layer (*11*–*14*), fluctuating or inaccurate metabolic rates (*5*, *15*–*17*), and vertical differences in the carbon isotopic composition of methane (*12*, *13*, *18*). Mass balance calculations that neglect lateral gas fluxes also fail to reproduce observed gas dynamics (*19*). While biological processes could partly explain some of these inconsistencies by modifying the reaction rates in Eq. 1 (*20*, *21*), several studies also point to a potential effect of advective exchange between littoral and pelagic regions (*5*, *8*, *11*, *12*, *16*, *19*, *22*). Frequently observed littoral-pelagic differences in gas concentrations (*9*, *12*, *14*, *23*, *24*) suggest that advective gas transport by cross-shore flows is possible. Therefore, scaling approaches assessing the relative contribution of advective transport to gas budgets must be developed (*7*), which requires a quantification of lateral flows responsible for advective gas fluxes.

Thermal siphon is a mechanism often evoked for advective lateral gas transport (*5*, *8*, *16*, *19*). This mechanism involves a two-limb (i.e., two-layer) convective circulation driven by differential cooling between littoral and pelagic zones. Surface cooling of the littoral slope region of a lake leads to a lateral density gradient that generates a downslope gravity current representing the lower limb of the circulation and a surface return flow representing the upper limb (Fig. 1) (*25*–*28*). The daily occurrence of convective circulation in lakes with expansive shallow nearshore regions (*29*) makes this process particularly relevant for littoral-pelagic exchange of water and solutes (*5*, *30*).

Variation in gas concentrations between nearshore and offshore areas, combined with downslope transport of littoral waters by the lower limb, will cause concentration anomalies in the offshore surface mixed layer. The upper limb drives additional lateral transport in the opposite direction, modifying concentrations in the littoral region. With a typical cross-shore velocity *U* ≈ 0.01 m s^−1^ (*29*), the advective transport by thermal siphons could exceed horizontal dispersion (*7*). Considering horizontal dispersion alone would thereby undermine littoral-pelagic fluxes in presence of convective circulation. Yet, even if convective circulation is a ubiquitous process that has been reported in numerous lakes (*26*, *29*–*33*), no studies have directly quantified its role in basin-scale redistribution of dissolved gases.

This study reports the results of field experiments that demonstrate lateral gas transport by convective circulation and interprets its impacts on gas flux estimates and concentration anomalies. The experiments were performed during autumn turnover in a eutrophic lake (Rotsee, Switzerland). We first showcase the efficiency of convective circulation in horizontal gas advection by tracking the spatial distribution of an inert tracer gas injected into the littoral region. Next, we examine the case of reactive gases (oxygen and methane) that exhibited two opposite lateral concentration gradients during our field experiment. Oxygen accumulated in shallow littoral zones was streamed offshore by the lower limb of the convective circulation, whereas methane produced in pelagic waters was transported onshore by the upper limb of convective circulation. These advective gas fluxes exceeded lateral fluxes typically assumed in gas budgets (*9*–*11*, *34*) by more than one order of magnitude and generated concentration anomalies in vertical gas profiles. The effectiveness of convective circulation for littoral-pelagic gas exchange demonstrates the need to integrate horizontal advective fluxes in lake ecosystem studies and models.

## RESULTS

### Cross-shore convective circulation driven by differential cooling

We quantified convective circulation driven by differential cooling from 6-day time series of currents and water temperature collected on 21 to 27 November 2020 in Rotsee (fig. S1). We focused on the cross-shore currents originating from the northeastern littoral plateau region and flowing along the lake’s main axis, *x* (Fig. 1A and fig. S1). We define littoral and pelagic as the regions where the lake is respectively shallower and deeper than the surface mixed layer (∼12 m deep) (*35*). Currents and water temperature were monitored in the littoral and pelagic regions, with mooring MT (thermal siphon mooring) at ∼4 m in depth and mooring MI (intrusion mooring) at ∼14 m in depth, respectively (Fig. 1, B and C). Low wind conditions prevailed during the study period (average wind speed ± SD of *U*_wind_ = 0.3 ± 0.4 m s^−1^ between 21 and 27 November; fig. S2B) and convection dominated over wind-driven motions (fig. S2C) (*36*). Differential cooling occurred continuously and led to lateral surface temperature gradients of ∂*T*/∂*x* ≈ 0.5°C km^−1^ with diel cycles (fig. S2D). Convective circulation formed at night, intensified in the morning, and weakened in the early afternoon (Fig. 1B) (*29*). Downslope gravity currents flowed at *U* ≈ 0.01 m s^−1^ and exhibited a thickness of *h* ≈ 1 m and vertical temperature gradient of ∂*T*/∂*z* ≈ 0.1°C m^−1^ with *z*, the vertical axis, directed upward. In the pelagic region, gravity currents streamed into the base of the surface mixed layer throughout the day (Fig. 1C) and reached the lake’s deepest point [background mooring (MB)], which was 800-m offshore from the plateau region (fig. S2F). These currents demonstrate the striking long-range capacity of convective circulation to physically connect the littoral zone with offshore zones of the lake. The discharge per unit width in both the upper and lower limbs of the circulation was *q* ≈ 0.01 m^2^ s^−1^, which flushed the 200-m-long and 1-m-deep plateau region in ∼6 hours. At that time of the year, convective circulation exchanged water daily between the littoral and pelagic regions of Rotsee and thereby had the potential to redistribute dissolved gases spatially. The turbulent Péclet number that relates the horizontal turbulent diffusion timescale to the advective timescale *Pe* = *UL*/*K_x_* (*4*, *7*) is equal to *Pe* ≈ 100 >> 1, with *K_x_* ≈ 0.1 m^2^ s^−1^ at length scales *L* ≈ 1000 m (*6*, *7*). This estimate indicates that advective transport by convective circulation is two orders of magnitude larger than lateral dispersion, which confirms that convective circulation must be considered in lake-wide mass balance approaches. The effect of this lateral exchange process on pelagic gas concentrations should be stronger during daytime, when convective circulation is more intense (*37*). This finding contrasts the assumption of horizontal convective transport being primarily a nocturnal process (*5*).

We use a 2-day subset of data from the November field campaign (24 to 26 November 2020) to analyze gas transport by convective circulation. Convective circulation intensified after sunrise on 25 November and persisted until the end of the campaign. This flow intensification led to a strong exchange between littoral and pelagic waters.

### Lateral transport of tracer gas

**Fig. 2.**
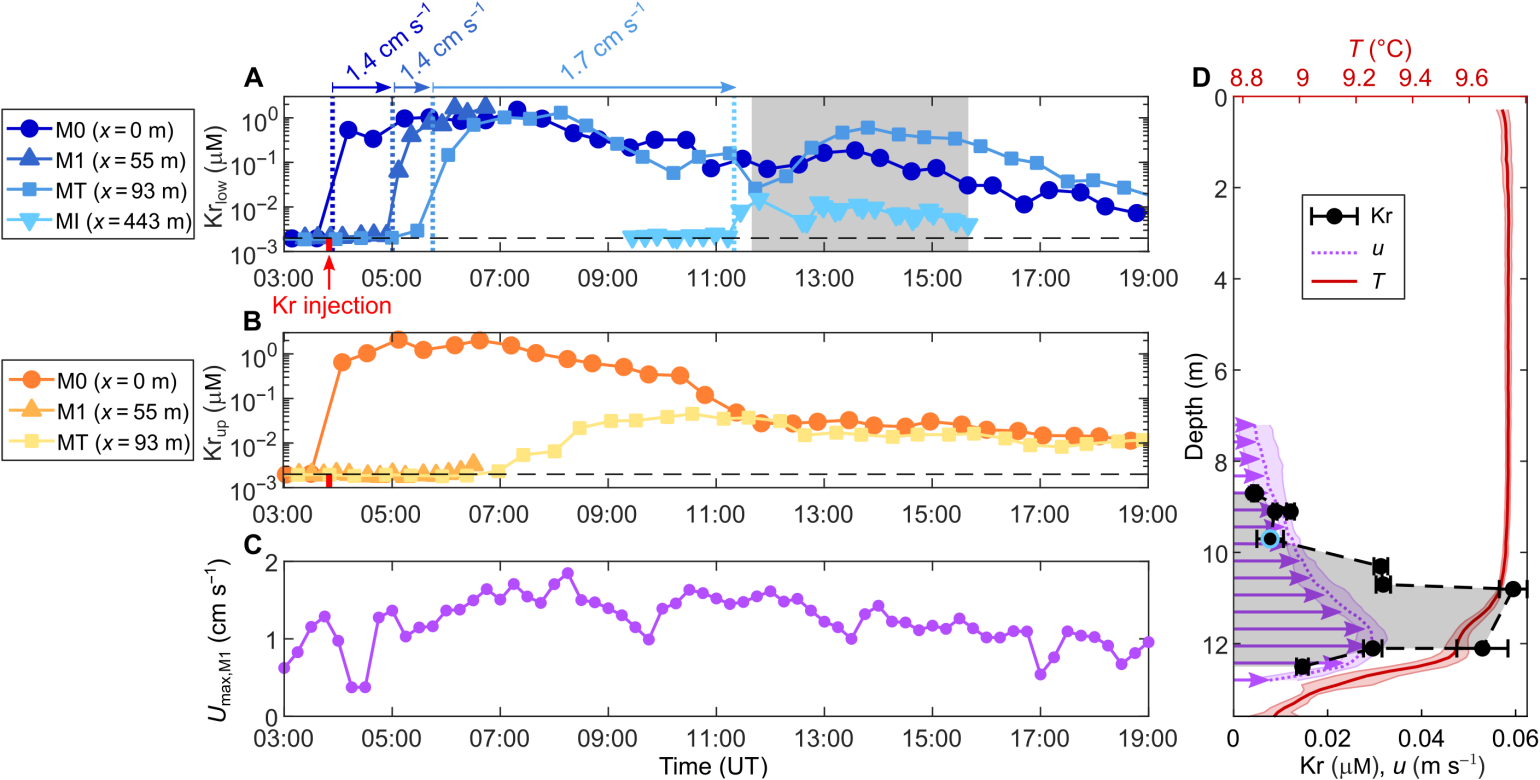
The lower circulation limb transports injected krypton offshore. (**A** and **B**) Time series of krypton concentrations at several locations along the cross-shore transect on 25 November in the lower (A) and the upper (B) circulation limbs. The red tick on the *x* axis in (A) indicates the time of krypton injection at M0. The horizontal black dashed line shows the background krypton concentration of ∼10^−3^ μM. Note the logarithmic scale on the *y* axis. Vertical dotted lines in (A) correspond to the arrival time of the krypton plume at each location used to estimate the plume velocity indicated on the upper *x* axis. (**C**) Time series of the maximal cross-shore velocity of the gravity current at M1 on 25 November. (**D**) Time-averaged vertical profiles of temperature (red), cross-shore velocity (purple), and krypton concentration (black dots) in the pelagic region at MI [11:40 to 15:40; gray area in (A)]. The SD is depicted with a shaded area on the velocity and temperature profiles and with horizontal error bars on the krypton profile. The blue circle in (D) indicates the depth of the MI time series shown in (A).

To determine whether convective circulation could efficiently transport gases from the littoral to the pelagic region, we performed a tracer experiment in which we injected an inert noble gas (krypton) into the littoral zone at M0 and tracked its concentration across the littoral and pelagic regions using two portable mass spectrometers (*38*) (Fig. 2). Noble gas tracer transport provided a physical quantification of gas dynamics free of biogeochemical reactions (*39*). After the injection on 25 November (03:50 UT), the krypton concentration at M0 reached ~1 μM, which is three orders of magnitude larger than the natural background concentration in equilibrium with the atmosphere (Fig. 2, A and B). A sharp increase in krypton concentration was detected at M1 and MT 1.1 and 1.9 hours after injection, respectively (Fig. 2A). This increase was confined to the downslope flow (0.5 m above sediment) and did not appear in the upper circulation limb (Fig. 2B). Thus, the lower circulation limb transported krypton and other dissolved species to deeper offshore waters. The slow increase in surface krypton concentrations observed in the littoral region at MT 3 hours after injection was due to vertical turbulent mixing between the two circulation limbs. The horizontal velocity of the krypton plume, estimated to be 1.4 cm s^−1^ from its arrival time at each station of the littoral region, matches the maximal cross-shore velocity of the downslope flow measured at M1 (Fig. 2C). The agreement between the arrival time of the krypton plume at each station and the expected arrival time based on the cross-shore flow velocity confirms that convective circulation drove the observed gas transport.

Krypton was further transported into the pelagic region, more than 400-m offshore from the injection point M0 (Fig. 2A). There, the krypton plume intruded the surface mixed layer at its base (Fig. 2D). The appearance of krypton in deep offshore waters demonstrates that convective circulation serves as an efficient physical bridge that connects littoral and pelagic zones.

### Littoral-to-pelagic downslope transport of oxygen

**Fig. 3.**
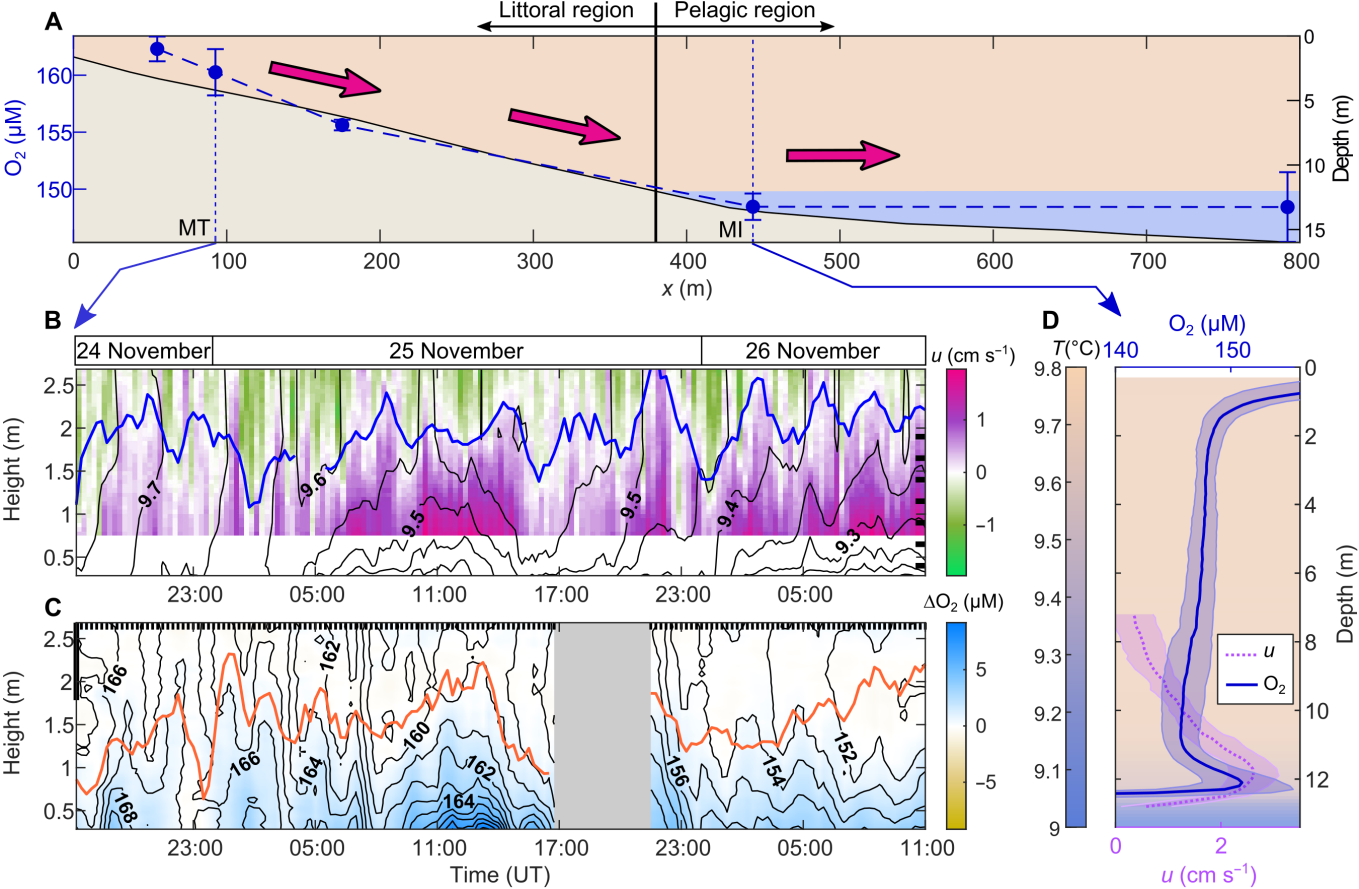
The lower circulation limb transports oxygen offshore. (**A**) Cross-shore oxygen concentration gradient in the mixed layer from CTDO profiles (25 November, 03:00 to 20:00). The concentration is averaged between 1.5 and 2.5 m in depth and between different profiles. Vertical error bars indicate the SD. The cross-shore topography is represented in light brown with yellow and blue areas corresponding to the epilimnion and hypolimnion, respectively. The vertical black line delimits the littoral and pelagic regions. Higher oxygen concentration nearshore leads to net offshore transport by the downslope gravity current (pink arrows). (**B** and **C**) Time series of cross-shore velocity (B) and oxygen concentration (C) in the littoral region at MT as a function of height above sediment from 24 to 26 November. The velocity time series in (B) are a subset of Fig. 1B, where black lines are 0.05°C spaced isotherms, black ticks on the right axis indicate the vertical location of the thermistors, and the blue line is the upper interface of the gravity current. The oxygen concentration in (C) is linearly interpolated from oxygen profiles collected every 15 min (vertical ticks on the upper *x* axis). The measurements were stopped between 17:00 and 21:00 on 25 November (gray area). The color map shows the oxygen concentration anomaly with respect to the mixed layer interior (1.5 to 2.5 m in depth, thick line on the left *y* axis). Black lines are 1 μM spaced isolines, and the orange line is the upper boundary of the bottom stratification (defined as ∂*T*/∂z > 0.01°C m^−1^ from temperature profiles). (**D**) Time-averaged vertical profiles of oxygen (blue) and cross-shore velocity (purple) in the pelagic region at MI (09:00 to 15:45). The shaded area around each profile indicates the SD. The background color map represents a time-averaged temperature profile similar to (A).

In addition to demonstrating downslope transport of an injected gas tracer, this research also investigated transport of naturally present gases. We collected conductivity-temperature-depth-oxygen (CTDO) profiles along a cross-shore transect on 25 November. Depth-averaged oxygen saturation in the mixed layer was ≤ 50% (fig. S5), and the concentration was 15 μM higher in the littoral region compared to the pelagic region (Fig. 3A and fig. S4). The depth-averaged cross-shore gradient of ∂O_2_/∂*x* = −30 μM km^−1^ (equivalent to −10% km^−1^; fig. S5) demonstrated natural conditions for offshore transport by the lower circulation limb.

Continuous measurements in the littoral region (MT) detected a persistent bottom layer with positive oxygen concentration anomalies of ΔO_2_ ≈ 3 μM relative to the surface layer and equivalent to a 1% saturation difference (Fig. 3C and fig. S5). This 1- to 2-m-thick layer was confined to the stratified downslope flow (Fig. 3B), as expected from downslope oxygen transport by the lower convective circulation limb. The lower circulation limb depth-averaged velocity of *U*_low_ ≈ 0.01 m s^−1^ generated a net advective oxygen flux into the pelagic region of *F*_O_2_,adv_ ≈ *U*_low_ ΔO_2_ ≈ 3 mol m−2 day−1. This advective flux exceeded by one order of magnitude the typical lateral flux due to horizontal dispersion F_O_2_,turb _ diff_ = −K_x_ ∂O_2_/∂x ≈ 0.1 mol m^−2^ day^−1^ with *K_x_* ≈ 0.1 m^2^ s^−1^ for length scales of ∼10^3^ m (*6*, *7*). Even with the highest estimate of *K_x_* = 0.7 m^2^ s^−1^ for length scales of ∼10^3^ m (*6*), the turbulent diffusive flux *F*_O_2_,turb_diff_ would still be twice lower than the observed advective flux *F*_O_2_,adv_.

As in the case of krypton, the gravity current transported oxygen across the pelagic region to form an oxic peak of 5 μM (equivalent to 1.5% saturation; fig. S5) at the base of the mixed layer (Fig. 3D). Advective oxygen transport by the lower circulation limb sufficed to overcome oxygen consumption (see Supplementary text) and explained the daytime positive concentration anomaly in the pelagic region.

### Pelagic-to-littoral surface transport of methane

**Fig. 4.**
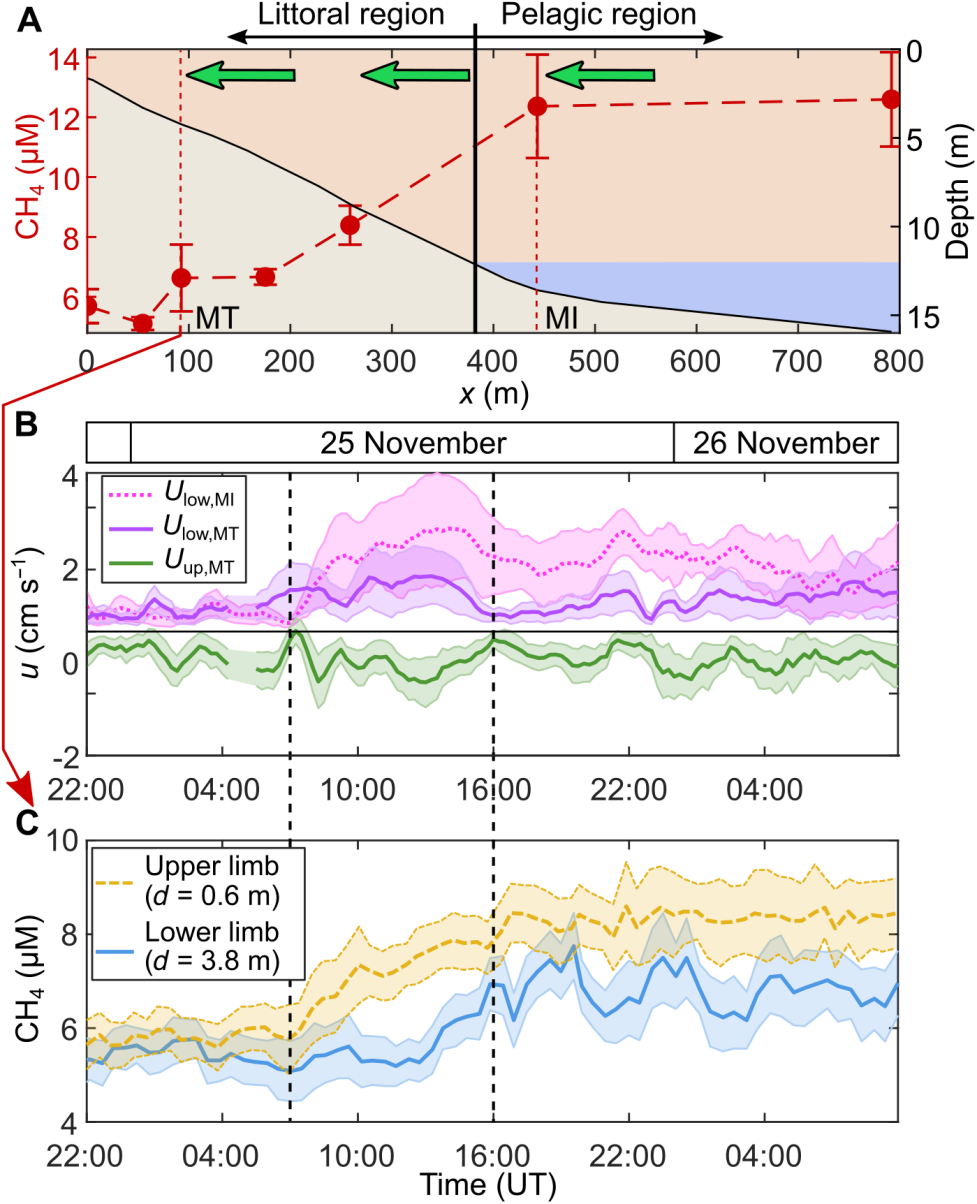
The upper circulation limb transports methane onshore. (**A**) Cross-shore gradient of methane concentrations in the mixed layer (24 November at 16:15 to 26 November at 11:00) measured by portable gas spectrometers. Concentrations are averaged over time and depth between the surface and the base of the mixed layer. Vertical error bars indicate the SD. The cross-shore topography is represented as in Fig. 3A. Higher methane concentrations near the lake center led to a net onshore transport by the surface return flow (green arrows). (**B**) Time series of depth-averaged cross-shore velocity in lower (purple, *U*_low_ > 0) and upper (green, *U*_up_ < 0) circulation limbs at MT and in the intrusion at MI (dotted line, *U*_low_ > 0) from 24 to 26 November. Shaded areas depict the SD. (**C**) Time series of methane concentration in the upper (yellow) and lower (blue) circulation limbs measured at MT by the portable gas spectrometer. Shaded areas depict the SD between five samples. The vertical dashed lines in (B) and (C) delimit the period of stronger circulation on 25 November at around 07:00 to 16:00.

Besides transporting gases concentrated nearshore toward the pelagic region via its lower limb, convective circulation should also transport gases concentrated offshore toward the littoral region via its upper limb (Fig. 1A). We examined pelagic-to-littoral transport by measuring methane concentrations with two portable mass spectrometers (*38*) along a cross-shore transect and continuously in the littoral region (MT). The mixed layer was strongly oversaturated with methane and showed concentrations of ~10 μM (Fig. 4A), consistent with previous measurements during autumn mixing events in Rotsee (*40*). Methane concentrations were two times higher in the pelagic region than in nearshore regions and exhibited a lateral gradient of ∂CH_4_/∂*x* = 15 μM km^−1^, likely due to the methane upward diffusion from the hypolimnion offshore. The inverse molar relationship between lateral methane and oxygen gradients ∂CH_4_/∂*x* ≈ −0.5 ∂O_2_/∂*x* was consistent with the stoichiometry of aerobic methane oxidation, suggesting that methane oxidation represents the main process generating the lateral oxygen gradient. While methane surface concentrations are typically higher in littoral areas than offshore during the stratified season (*9*, *12*, *14*, *41*), the lateral methane gradient can reverse during autumn turnover in eutrophic lakes when vertical convective mixing brings methane-enriched hypolimnetic waters to the surface (*24*). This reversed gradient allowed us to verify the onshore transport by the upper circulation limb.

We compared methane concentration time series from the littoral region (MT) before and after the flow intensification on 25 November (Fig. 4, B and C). When convective circulation intensified in both the littoral and pelagic regions (07:00 to 16:00), the surface methane concentration at MT increased by ΔCH_4_ ≈ 2 μM. This temporal increase is consistent with surface methane transport from MI to MT by the upper circulation limb. Methane transport causes a net onshore flux of *F*_CH_4_,adv_ ≈ ∣*U*_up_∣ΔCH_4_ ≈ 2 mol m^−2^ day^−1^ with ∣*U*_up_∣ ≈ 0.01 m s^−1^. As for oxygen, the methane advective flux is one order of magnitude larger than the lateral flux assumed from the horizontal turbulent diffusion coefficient of *K_x_* ≈ 0.1 m^2^ s^−1^ for length scales of ∼10^3^ m (*9*). Once the flow became steady, the surface methane concentration at MT remained ∼8 μM, i.e., 50% lower than values observed in the pelagic zone at MI. Differences likely reflect methane losses by oxidation and atmospheric exchange between MI and MT (see Supplementary text). Our data confirms the onshore surface methane transport across the entire littoral region up to M0 (fig. S6).

## DISCUSSION

Our study demonstrates that convective circulation plays a substantial role in littoral-pelagic connectivity by transporting and thus redistributing dissolved gases laterally on a daily basis. The injection of a noble tracer gas allowed us to rigorously link physical water transport with gas exchange, because the latter can be masked by the dynamics and reactions of naturally occurring gases. We further showed that the observed transport not only is limited to offshore transport via the lower limb but also includes onshore transport via the upper limb. The resulting advective fluxes of oxygen and methane were one order of magnitude larger than horizontal dispersion fluxes typically used to model the lateral transport of gases (*9*–*11*), which emphasizes the need to include horizontal advection in mass budgets (Eq. 1). Lateral advection overcame consumption rates (see Supplementary text) and modified vertical concentration profiles not only in the pelagic but also in the littoral regions. Although the magnitude of concentration gradients and lateral fluxes remain site specific, the transport mechanisms presented here are ubiquitous among lakes with shallow littoral areas (*25*, *26*, *30*–*33*). These lakes are often characterized by lateral gradients of dissolved oxygen, carbon dioxide, and methane (*9*, *12*, *14*, *23*, *24*), suggesting that convective circulation frequently redistributes gases during calm conditions.

Despite the limited period of our gas experiment in late autumn, we expect convective circulation to transport gases in Rotsee from late summer to winter (*29*), as long as lateral concentration gradients are present and winds are light. Because nighttime cooling is one order of magnitude greater in summer, the summer lateral gas transport should be twice faster, yet less frequent, than in autumn [see figure 6 in (*29*)]. The faster transport may generate larger pelagic gas anomalies in summer if coupled with strong lateral concentration gradients. These anomalies would, however, not persist during warm periods without convective circulation and would not occur in spring when surface cooling is weak. Wind-driven flows may suppress or enhance lateral transport by convective circulation (*36*) and may also contribute to advective gas transport. This could be the case in Rotsee in spring and early summer when wind-driven flows dominate lateral exchange (*29*). Concurrent measurements of lateral transport and gas dynamics in different seasons are required to relate convective circulation to typical gas anomalies.

The lateral gas exchange described in this study is not gas specific but depends rather on lateral concentration gradients. Increasing methane concentrations toward the littoral region with gradients of ∣∂CH_4_/∂*x*∣ ≈ 0.1 − 10 μM km^−1^ have been reported in lakes (*12*, *14*, *24*, *41*) and observed in Rotsee during the summer-stratified period (fig. S8). This implies substantial offshore methane transport. This transport can generate a methane peak at the base of the mixed layer when advection overcomes losses as observed for krypton and oxygen by this research. From typical methane oxidation rates of *R*_ox,CH_4__ = 0.01 − 1 μmol CH_4_ liter^−1^ day^−1^ (*18*, *34*, *42*, *43*) and cross-shore velocities of *U*_low_ ≈ 0.01 m s^−1^, the minimum lateral gradients required to overcome oxidation rates would be ∣∂CH_4_/∂*x*∣ ≈ *R*_ox,CH_4__/*U*_low_ ≈ 0.01 − 1 μM km^−1^. These fall within an order of magnitude of observed methane gradients. Convective circulation is thereby able to generate methane peaks in the pelagic region of lakes if (i) differential cooling occurs for a sufficient period (*29*) and (ii) lateral methane gradients are present (see Supplementary text). In this case, convective circulation should be included in methane budgets in addition to other biological and physical processes responsible for vertical methane peaks.

Lateral oxygen concentration gradients vary depending on differences in metabolic rates between littoral and pelagic regions (*5*, *8*, *22*, *23*, *44*). Assuming a nocturnal decrease and a daytime increase in oxygen concentration toward the shore during summer stratification (*5*), morning transport of oxygen-depleted water by the lower convective circulation limb would bias estimates of pelagic metabolic rates by the diel oxygen method. These biases would appear mostly during daytime hours experiencing maximal lateral transport. This contrasts the classic assumptions of nocturnal increase in pelagic oxygen concentration by differential cooling (*5*, *16*). The daily periodicity of thermal siphons precludes removal of their systematic effects on metabolic rate estimates by the standard multiple day average procedure (*17*).

We encourage future biogeochemistry studies to systematically determine the potential contribution of convective circulation to lake-wide mass budgets by comparing timescales for lateral advection, horizontal turbulent diffusion, and reaction (fig. S9). Lateral advection dominates the dynamics of dissolved constituents in Eq. 1 when the turbulent Péclet number *Pe* = *UL*/*K_x_* > 1 and the advective Damköhler number, i.e., the ratio between the advective timescale to the reaction timescale, *Da* = μ*L*/*U* < 1 (*4*, *7*). In the previous equations, *U* is the lateral velocity scale, *L* is a characteristic length scale of the lake, *K_x_* is the horizontal turbulent diffusion coefficient estimated from *L* (*6*), and μ is the reaction rate of the dissolved substance of interest (see Supplementary text). The lateral velocity scale of convective circulation during low-wind conditions can be estimated from meteorological forcing and topography (fig. S10) (*29*). It should be corrected to account for cross-shore winds (*36*).

By demonstrating the role of convective circulation for lateral gas transport, results reported here reveal that shallow littoral regions can strengthen the littoral-pelagic connectivity and modify the basin-scale distribution of dissolved gases. Two-dimensional model frameworks integrating lateral advective transport should be developed to improve the estimation of gas production and consumption rates. Systematic quantification of lateral fluxes in gas budgets can further elucidate the overall physical, chemical, and biological dynamics of aquatic ecosystems.

## MATERIALS AND METHODS

### Study site

Rotsee is a small eutrophic peri-alpine lake in Central Switzerland (47°4′29″N, 8°19′29″E; elevation of 419 m above sea level; surface area of 0.5 km^2^; mean and maximum depths of 9 and 16 m, respectively). It consists of an elongated wind-sheltered basin (2.5 km long, 0.2 km wide). The main inflow at the southwestern end and the outflow at the northeastern end (fig. S1) have a low discharge of ∼0.1 m^3^ s^−1^. The 200-m-long and 1-m-deep plateau region at the northeastern end of the lake (fig. S1) fosters convective circulation at a daily frequency, mostly in autumn, in response to differential cooling (*29*). The anoxic hypolimnion of Rotsee accumulates methane during the stratified season in summer. Hypolimnetic waters with low oxygen and high methane concentrations are entrained into the pelagic epilimnion during autumn lake turnover, where methane is both oxidized and released to the atmosphere (*18*, *40*, *43*, *45*).

### Physical measurements

We defined the *x* axis as the preferential flow direction along the lake thalweg, from the shore to the lake center, and we set its origin at the end of the plateau region (point M0; Fig. 1A). To monitor the cross-shore circulation originating from the northeastern plateau region, we deployed four vertical thermistor arrays (M1, MT, M2, and MI) along the *x* axis, from 21 to 27 November 2020 (Fig. 1A and fig. S1). The RBR thermistors (TR-1050, TR-1060, Solo-T) collected water temperature at 1–10 s sampling intervals with a vertical resolution of 0.25 to 0.5 m (see depth locations for each mooring in Figs. 1 and 3 and figs. S2 and S3). Water flow velocity was continuously measured as a function of depth by bottom-moored upward-looking acoustic doppler current profilers (ADCPs) at M1 (Nortek Aquadopp Profiler, 2 MHz, profiling 0.23 to 1.76 m above sediment with 0.03-m vertical resolution), MT (Teledyne RD Instruments WorkHorse, 1200 kHz, profiling 0.75 to 3.80 m above sediment with 0.05-m vertical resolution), and MI (Teledyne RD Instruments WorkHorse, 600 kHz, profiling 0.8 to 6.4 m above sediment with 0.1-m vertical resolution). The three ADCPs measured in pulse-to-pulse coherent mode and provided burst-averaged three-dimensional velocity data every 15 min. An additional thermistor array (MB, Vemco Minilog II-T loggers; 1-m vertical resolution and 2-min sampling interval) monitored the background thermal structure near the deepest point. A WxPRO Campbell weather station installed on the lake shore near the plateau region (47°4′34″N, 8°19′38″E) provided local meteorological forcing during the entire measurement period (atmospheric pressure, air temperature, wind speed and direction, incoming shortwave radiation, and relative humidity with a 10-min temporal resolution). Further details on the physical measurements are given in (*29*).

### Dissolved gas measurements during the 2-day campaign

We performed a 2-day campaign from 24 to 26 November 2020 to quantify the lateral gas transport in the two limbs of the convective circulation. Two portable mass spectrometers miniRUEDI (Gasometrix, Switzerland) measured the krypton and methane [among other gases (*38*, *39*)] concentration on site with an analytical uncertainty of 1 to 3% (*38*). Each spectrometer received lake water pumped from specific depths. The inflowing water was filtered (pore size of 20 μm) before entering a gas-equilibrium membrane contactor, where dissolved gases were transferred to the air phase and brought to the mass spectrometer through a capillary. One spectrometer was installed on a moored rowing platform at MT. It continuously sampled the littoral region at M0 (*x* = 0 m, 1.6 m deep) at 0.9 and 1.3 m in depth and at MT (*x* = 90 m, 4.3 m deep) in the upper (0.6 m deep) and lower (3.8 m deep) limbs of the circulation (Fig. 1A and table S1). Lake water was collected at each of these four locations by a submersible pump and carried to the spectrometer via a polyvinyl chloride tube. After flowing through the membrane contactor, the water was pumped to the southeastern shore to prevent any contamination of the measurements at MT. The second spectrometer sampled the five other stations (M1, M2, M3, MI, and MB) from a boat (Fig. 1A and table S1). The samples were continuously collected at each mooring for several hours before moving to another location. Two submersible pumps, both combined with a RBRduet T.D logger for pressure measurements, were lowered at two different depths for each station. One pump was kept at ∼0.5 m below the surface (upper limb of the circulation), and the other was used to profile different depths (M3 and MI) or the base of the mixed layer only (M1, M2, and MB; lower limb of the circulation). The outflowing water of the membranes was pumped back to the sampling depth. Both spectrometers sampled the different inlets (depths) one after the other. The same inlet was sampled every 14 min on the boat and every 28 min on the platform. These sampling intervals were longer than the water residence time in the tube (∼7 min between M0 and the moored spectrometer). For each inlet, average gas concentrations were computed from five repeated samples, spaced 17 s apart. The measurements were calibrated by sampling a standard gas (air enriched with 1% CH_4_ and 1% CO_2_) every hour. In addition, cross-calibration between the two spectrometers was performed from measurements at nearby locations. At each location, the average methane concentration in the mixed layer was computed by averaging the concentrations at the surface and at the mixed layer base.

We collected 243 CTDO profiles (Sea & Sun Technology, CTD 60 M) at an interval of ∼15 min from the boat at M1, M2, MI, and MB (same periods as for the spectrometer; table S1) and from the platform at MT (24 November at 17:00 to 26 November at 11:00, with an interruption between 17:00 and 21:00 on 25 November). Both profilers were equipped with an optical oxygen sensor (±2% accuracy and estimated precision of 0.1% saturation or 0.3 μM) that measured dissolved oxygen concentration. For each profile, we calculated the average oxygen concentration in the mixed layer interior, between 1.5 and 2.5 m in depth, to remove the oxygen peaks at the surface (atmospheric input) and at the bottom (lateral transport) (Fig. 3D).

### Gas tracer experiment

We performed a gas tracer experiment on 25 November by injecting krypton in the littoral region at M0 and tracked its offshore transport with both spectrometers. A 10-liter krypton bottle (∼60 moles of atmospheric Kr at 142 bar) was installed at the shore and connected to an impermeable pipe that reached M0. A diffuser, made of a 20-m-long porous pipe, was placed on the sediment, perpendicularly to the *x* axis, with M0 in its middle. The diffuser produced bubbles, which mixed the gas vertically across the water column. On 25 November, we started the injection at 3:50 UT by opening the krypton bottle. The ∼60 moles of Kr was injected within ∼4 hours.

### Velocity data analysis

The velocity data collected by the ADCPs was projected onto the *x* axis (56° angle from north) to obtain the cross-shore velocity *u*. The depth of the interface *z*_int_ between the two circulation limbs was found from the stagnation point *u* = 0. Depth-averaged velocities of the lower and upper limbs were calculated as $U_{\text{low}}\left(t\right)=\frac{1}{z_{\text{int}}-z_{\text{sed}}}\int_{z_{\text{sed}}}^{z_{\text{int}}}u\left(z,t\right)\mathrm{dz}$ and $U_{\text{up}}\left(t\right)=-\frac{1}{z_{\text{int}}}\int_{z_{\text{int}}}^{0}u\left(z,t\right)\mathrm{dz}$, respectively, with *z*_sed_ ≈ −4 m the lake depth at MT.

## Supplementary Materials

### This PDF file includes:

Supplementary Text Figs. S1 to S10 Table S1 References
