# Supplementary material for: Lake surface cooling drives littoral-pelagic exchange of dissolved gases
Source: Sci Adv. 2024 Jan 24;10(4):eadi0617. doi: 10.1126/sciadv.adi0617 (PMC10807797; doi:10.1126/sciadv.adi0617)
Supplement: Supplementary file 1 — Supplementary Text Figs. S1 to S10 Table S1 References [file sciadv.adi0617_sm.pdf]

Science Advances
AAAS

Supplementary Materials for

Lake surface cooling drives littoral-pelagic exchange of dissolved gases

Tomy Doda *et al.*

Corresponding author: Tomy Doda, tomy.doda@eawag.ch; Damien Bouffard, damien.bouffard@eawag.ch

Sci. Adv. **10**, eadi0617 (2024)
DOI: 10.1126/sciadv.adi0617

This PDF file includes:

Supplementary Text
Figs. S1 to S10
Table S1
References

Supplementary Text

Estimation of oxygen consumption during lateral transport

According to the potential oxygen consumption rate of $R_{ox,O_2} = 30$ µmol O_2 L^{-1} d^{-1} in Rotsee (*46*), the amount of oxygen consumed during the six-hour travel time τ from the littoral (M1) to the pelagic region (MI) (Fig. 2A) amounts to $R_{ox,O_2} \cdot \tau = 9$ µmol L^{-1}. This estimate represents $\sim$ 60 % of the lateral oxygen concentration difference of 14 µmol L^{-1} between sampling sites M1 and MI (Fig. 3A) and suggests an oxygen increase of ~ 5 µmol L^{-1} at the base of the mixed layer which is consistent with our observations (Fig. 3D). If we assume first-order kinetics for the oxygen consumption, $R_{ox,O_2} = \mu_{O_2} C_{O_2}$ where $C_{O_2} \approx 150$ µmol L^{-1} is the oxygen concentration and $\mu_{O_2} = R_{ox,O_2}/C_{O_2} \approx 0.2$ d^{-1} is a decay rate. The advective Damköhler number relating the advection timescale to the reaction timescale $Da_{O_2} = \mu_{O_2} L/U$ (*4, 7*) is on the order of $Da_{O_2} \approx 0.1 < 1$ at length scales of $L \approx 1000$ m and with the velocity of the convective circulation $U \approx 0.01$ m s^{-1}. This estimate confirms that advective transport overcomes oxygen consumption (*7*).

Estimation of methane losses during lateral transport

The amount of methane oxidized during the eight-hour travel time τ from the pelagic region to MT (300 m distance travelled at 0.01 m s^{-1} velocity) is $\tau \cdot R_{ox,CH_4} \approx 2$ µmol L^{-1} according to typical oxidation rates of $R_{ox,CH_4} = 5$ µmol CH_4 L^{-1} d^{-1} in autumn in Rotsee (*18, 41*). Atmospheric exchange additionally decreased the methane concentration by $\tau \cdot F_{atm}/d \approx 0.5$ µmol L^{-1}, with $F_{atm} \approx 0.1$ µmol CH_4 m^{-2} s^{-1} the atmospheric methane flux in Rotsee estimated from meteorological data (fig. S7) and measured in autumn (*40*), and $d = 7$ m the average lake depth between MT and the pelagic region. Based on the initial concentration difference of 6 µmol L^{-1} between MT and MI (Fig. 4A), the total methane loss of ~ 3 µmol L^{-1} suggests an increase of surface methane concentration of ~ 3 µmol L^{-1} at MT, which agrees well with the observed ΔCH_4 (Fig. 4C). The advective Damköhler number is given by $Da_{CH_4} = \mu_{CH_4} L/U$ (*4, 7*), where $U \approx 0.01$ m s^{-1} is the velocity of the convective circulation, $\mu_{CH_4} = (R_{ox,CH_4} + F_{atm}/d)/C_{CH_4} \approx 0.5$ d^{-1} is the methane decay rate and $C_{CH_4} \approx 13$ µmol L^{-1} is the methane concentration offshore. The Damköhler number is $Da_{CH_4} \approx 0.5 < 1$ at length scales of $L \approx 1000$ m, which confirms that advective transport overcomes methane consumption.

Contribution of convective circulation to pelagic methane peaks

In Lake Hallwil and Lake Stechlin, where methane peaks of $\Delta CH_4 \approx 0.1 - 1$ µmol L^{-1} in the oxic layer have been reported at a distance of $L \sim 1$ km from the shore, methane oxidation rates and littoral-pelagic concentration differences were $R_{ox,CH_4} \approx 0.01$ µmol CH_4 L^{-1} d^{-1} and $\Delta_x CH_4 \approx 0.1 - 1$ µmol L^{-1} (gradient of $|\Delta_x CH_4/L| \approx 10^{-1} - 10^{0}$ µmol L^{-1} km^{-1}), respectively (*8, 10, 13*). According to our estimates from Rotsee (velocity of the lower circulation limb $U_{low} \approx 0.01$ m s^{-1}), we expect convective circulation to generate a peak of $\Delta CH_4 = \Delta_x CH_4 - R_{ox,CH_4} L/U_{low} \approx 0.1 - 1$ µmol L^{-1}, if vertical mixing between the two limbs is neglected. This estimate agrees with the orders of magnitude of reported ΔCH_4, indicating that convective circulation is at least as important as other chemicophysical processes generating methane peaks.

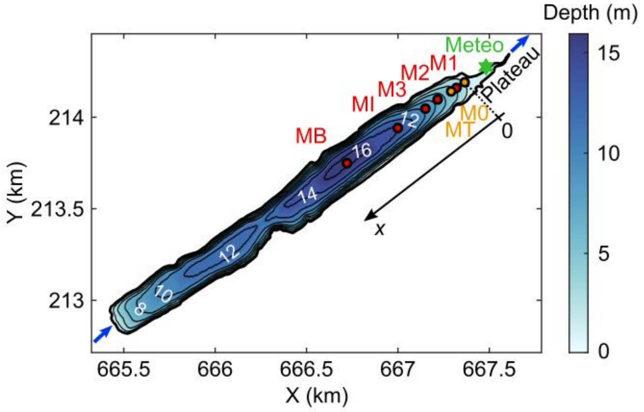

Fig. S1. Bathymetric map of Rotsee. Red and orange dots indicate the location of the seven measurement stations. Continuous measurements of dissolved gases were collected at M0 and MT (purple dots). The green star depicts the weather station. Blue arrows show the small inflow and the outflow. Black lines with white depth labels are 2 m spaced isobaths.

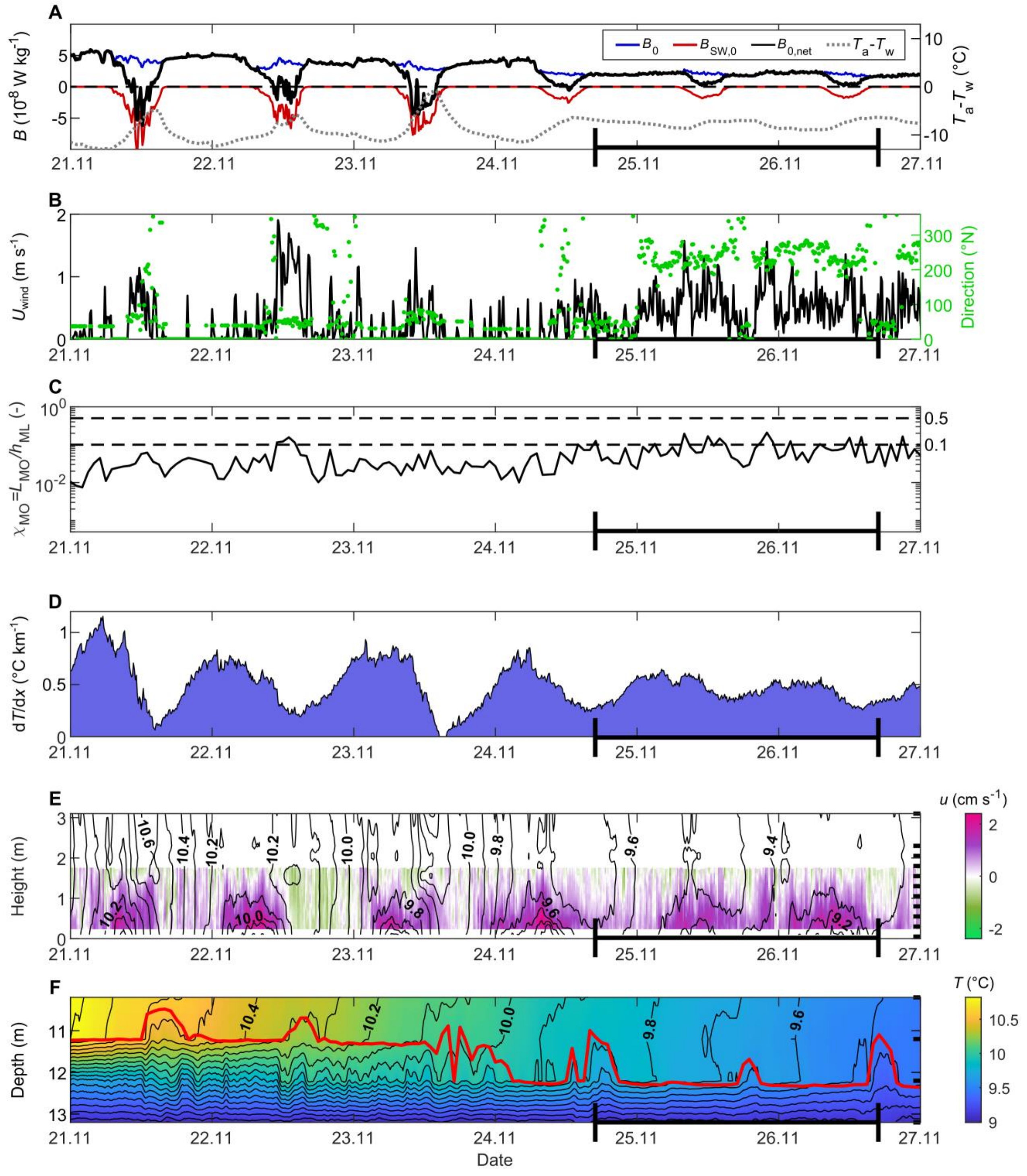

Fig. S2. Forcing conditions and convective circulation. Six-day time series of surface buoyancy fluxes and air-water temperature differences **(A)**, wind speed and direction at 3 m height **(B)**, dimensionless Monin-Obukhov length **(C)**, lateral temperature gradients between M1 and MI **(D)**, cross-shore velocity at M1 **(E)** and water temperature at MB **(F)**, on 21-27 November 2020 (same period as in Fig. 1, B and C) with the gas experiment period on 24-26 November scaled in bold on the x-axis of each plot. **(A)** The non-penetrative surface buoyancy flux (B_0), the shortwave surface buoyancy flux ($B_{SW,0}$) and the net surface buoyancy flux ($B_{0,net} = B_0 + B_{SW,0}$) were computed as $B_0 = \alpha g H_{Q,0}/\rho C_{p,w}$ and $B_{SW,0} = \alpha g H_{SW,0}/\rho C_{p,w}$ where $H_{Q,0}$ is the sum of sensible heat, latent heat and longwave radiation fluxes, $H_{SW,0}$ is the incoming shortwave radiation corrected for the

wavelengths range of the pyranometer and for the water surface albedo, α is the thermal expansivity, g is the gravitational acceleration, ρ is the water density and $C_{p,w}$ is the specific heat capacity of water. Each heat flux component was calculated following Fink et al. (2014) (*47*) except the incoming longwave radiation directly measured at the Lucerne weather station, 4 km from Rotsee, as in Doda et al. (2022) (*29*). Destabilizing fluxes are positive (cooling), whereas stabilizing fluxes are negative (heating). Net surface cooling ($B_{0,net} > 0$) occurred over the entire experiment period (24-26 November). The grey dotted line corresponds to the air-water temperature difference $T_a - T_w$. **(B)** The black line and the green dots show the 10 min wind speed and direction, respectively. **(C)** The two horizontal dashed lines indicate the wind-convection interaction regime $0.1 \leq \chi_{MO} \leq 0.5$ (*36*). **(D)** The lateral temperature gradients were calculated from mixed layer averaged temperature at M1 and MI (390 m apart). Positive temperature gradients correspond to differential cooling. **(E)** The cross-shore velocity is represented as in Fig. 1, B and C. **(F)** The red line depicts the mixed layer depth, defined as in Doda et al. (2022) (*29*). The daily thickening of the stratified bottom layer in the afternoon (rise of the red interface) indicates intrusions of cold water from convective circulation. Black lines in **(E-F)** are 0.1°C spaced isotherms, from linearly interpolated temperature between each thermistor (black ticks on the right y-axis).

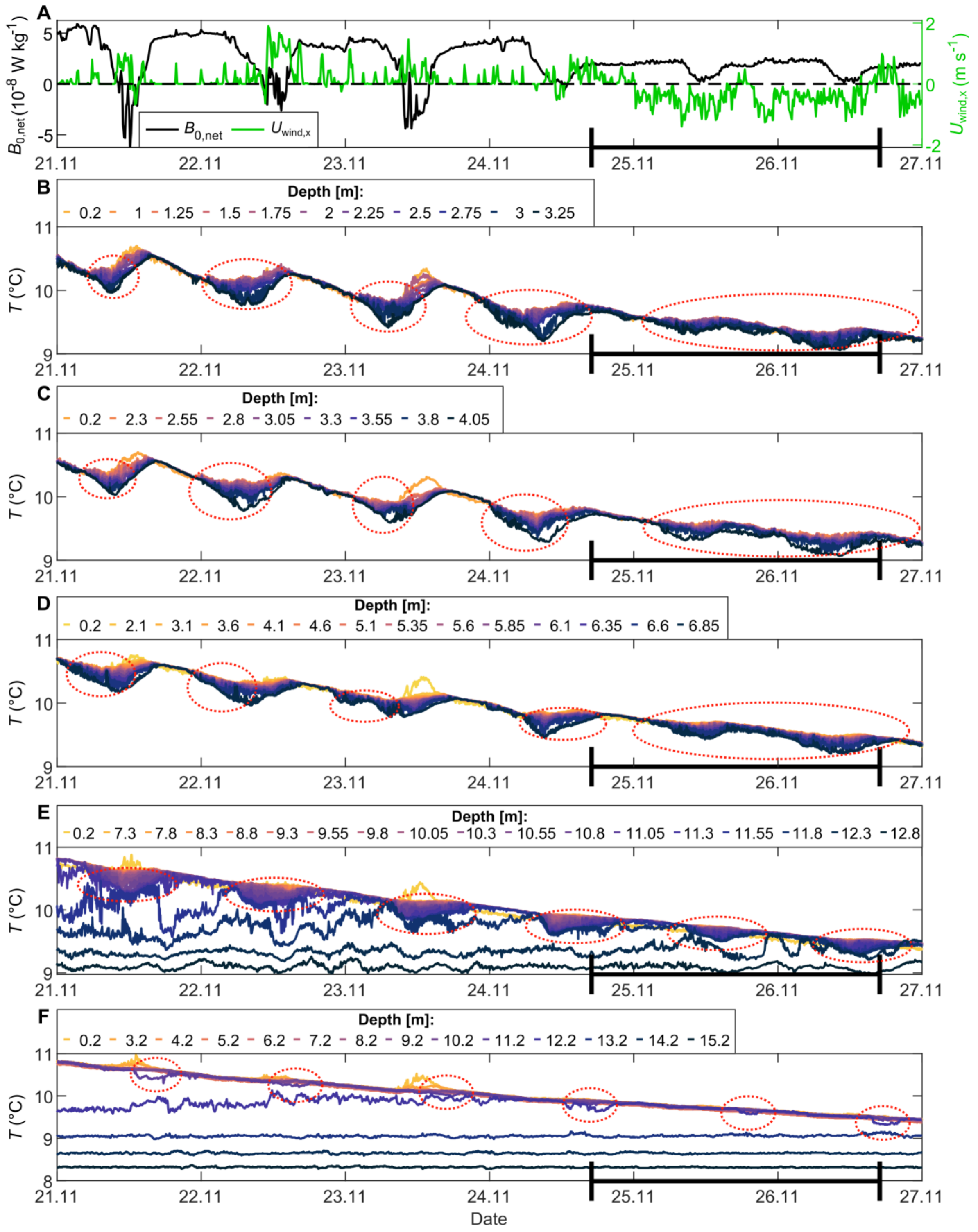

Fig. S3. Forcing conditions and temperature time series at each mooring. Six-day time series on 21-27 November 2020 (same period as in Fig. 1, B and C) with the gas experiment period on 24-26 November scaled in bold on the x-axis of each plot. **(A)** Net surface buoyancy flux (black line) and wind speed along the x-axis (green line). **(B** to **F)** Temperature time series at each sensor depth at M1 **(B)**, MT **(C)**, M2 **(D)**, MI **(E)** and MB **(F)**. The red dotted circles indicate a stratified layer at the base of the mixed layer associated with convective circulation.

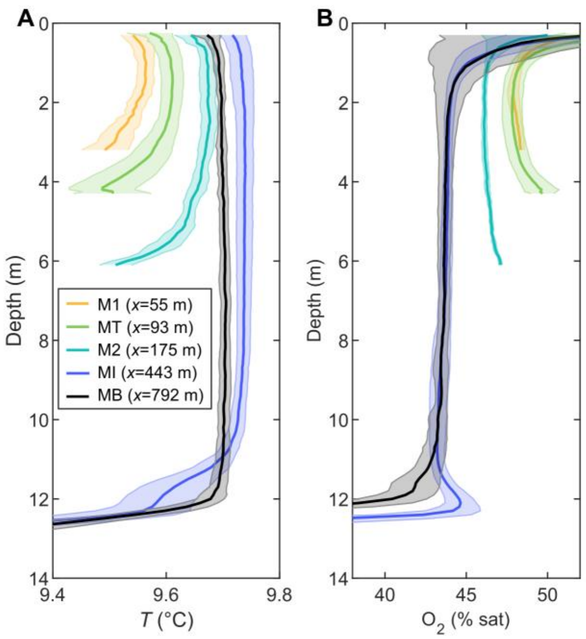

Fig. S4. Cross-shore variability of temperature and oxygen saturation. Temporally averaged profiles of temperature **(A)** and oxygen saturation **(B)** at five locations along the cross-shore transect (25 November, 03:00-20:00). Shaded areas depict the standard deviation. **(A)** The lateral mixed layer temperature gradient due to differential cooling between M1 and MI (littoral region) is $\partial T/\partial x \approx 0.4$ °C km^{-1}. The bottom stratification induced by the downslope gravity current is visible at M1, MT and M2. The intrusion at MI creates a cold layer at the base of the mixed layer that is not observed in the temporally averaged profile at MB. **(B)** The lateral mixed layer oxygen gradient between M1 and MI (littoral region) of $\partial O_2/\partial x \approx -10$ % km^{-1} is the same as in fig. S5A. The downslope oxygen transport increases the oxygen concentration near the bottom at M1, MT and M2. The intrusion at MI creates an oxic peak at the base of the mixed layer that is not observed at MB.

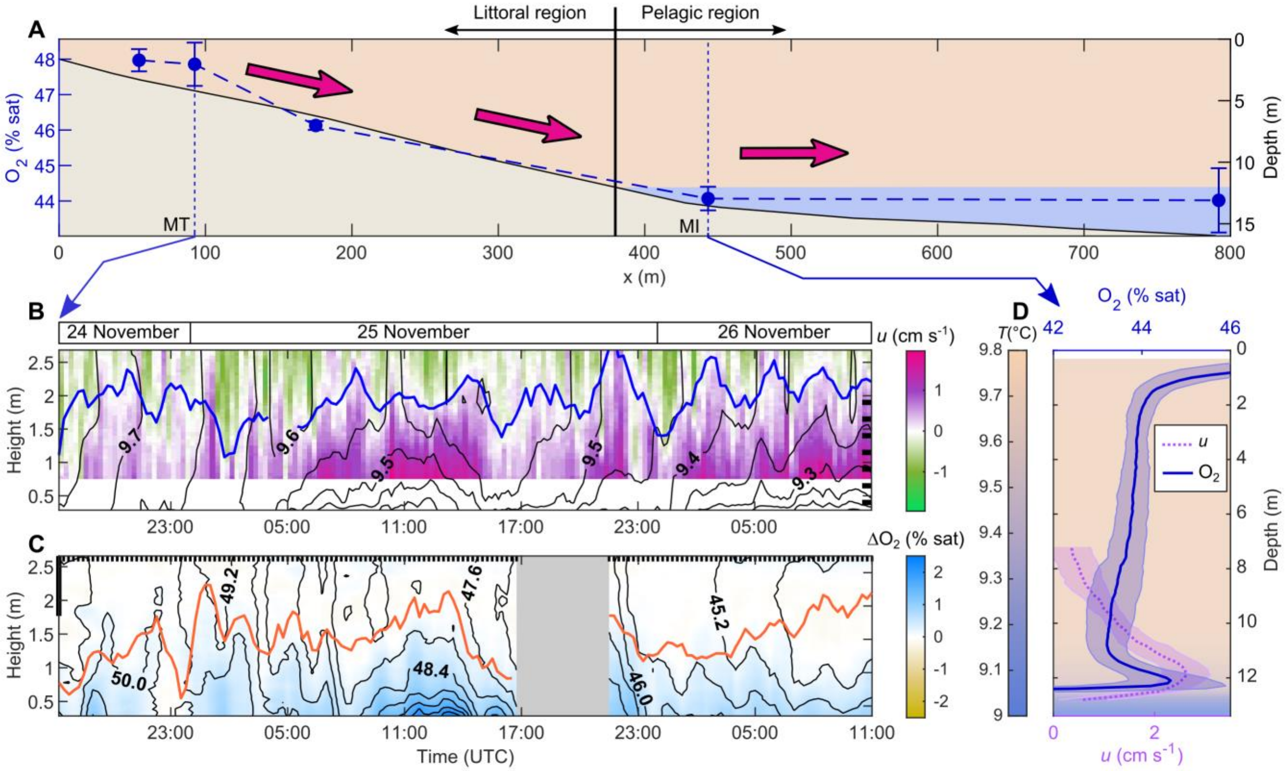

Fig. S5. Oxygen transport by the lower circulation limb expressed in oxygen saturation. Same figure as Fig. 3 with percentage of oxygen saturation instead of oxygen concentration.

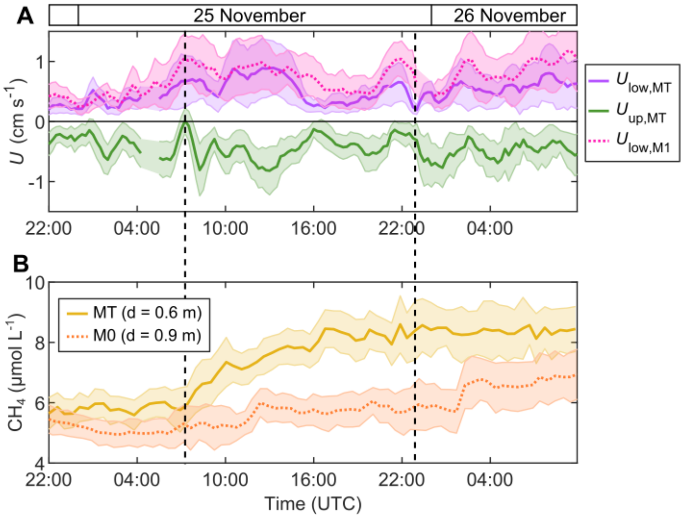

Fig. S6. Surface transport of methane across the littoral region. (A) Time series of depth-averaged cross-shore velocity in the lower limb (purple, $U_{low} > 0$) and in the upper limb (green, $U_{up} < 0$) of the circulation at MT, and in the lower limb at M1 (dotted line, $U_{low} > 0$), from 24 to 26 November. Shaded areas depict the standard deviation. **(B)** Time series of methane concentration in the upper limb of the circulation at MT (solid line) and M0 (dotted line). Shaded areas depict the standard deviation between five samples. The vertical dashed lines show two flow intensification events in the littoral region on 25 November, after 07:00 and after 23:00. Both events enhanced the surface methane transport from MT to M0, as shown by the sharp concentration increase at M0 4-5 h after each flow intensification.

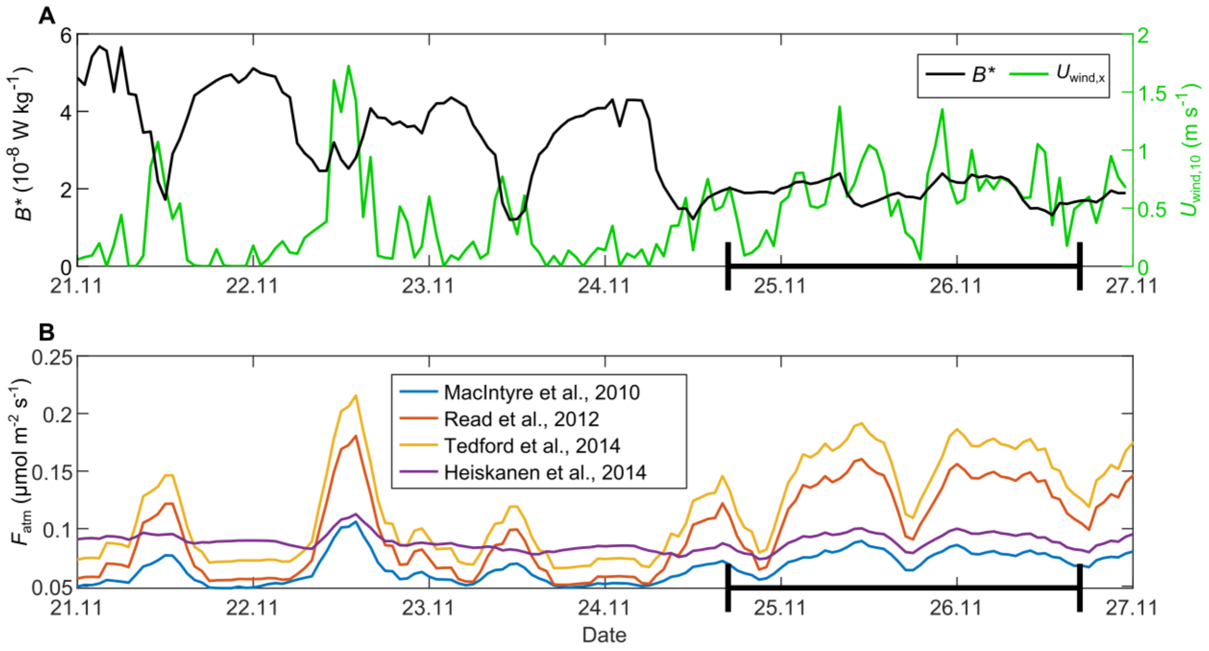

Fig. S7. Estimation of air-water methane fluxes. Six-day time series on 21-27 November 2020 (same period as in Fig. 1, B and C) with the gas experiment period on 24-26 November scaled in bold on the x-axis of each plot. **(A)** Effective buoyancy flux in the mixed layer and wind speed $U_{\text{wind},10}$ at 10 m height. The effective buoyancy flux is computed as $B^* = \alpha g H^* / \rho C_{p,w}$ where $H^* = H_{Q,0} + H_{SW,0} + H_{SW}(-h_{ML}) - 2/h_{ML} \int_{-h_{ML}}^{0} H_{SW}(z) dz$ (*48*), $H_{SW}(z) = H_{SW,0} \exp\,(k_d z)$, $k_d = 0.2$ m^{-1} is the light attenuation coefficient in Rotsee in late autumn and h_{ML} is the mixed layer depth computed as in Doda et al. (2022) (*29*). **(B)** Air-water methane flux estimated with four methods that include wind and convection effects on surface turbulence. The atmospheric flux was calculated as $F_{atm} = k_{CH_4}(C_{CH_4,w} - C_{CH_4,sat})$, where k_{CH_4} is the piston velocity, $C_{CH_4,w} \approx 12$ µmol L^{-1} is the surface methane concentration and $C_{CH_4,sat}$ is the methane concentration in equilibrium with the atmosphere (1.88 ppm). The piston velocities [m s^{-1}] from Read et al. (2012) (*49*), Heiskanen et al. (2014) (*50*) and Tedford et al. (2014) (*51*) were estimated with the surface renewal model as $k_{CH_4} = c_1[(c_2 B^* + c_3 u_*^3/(\delta\kappa))\nu]^{1/4}(Sc_{CH_4})^{-1/2}$, where u_* is the friction velocity calculated from $U_{w,10}$ as in Read et al. (2012) (*49*), $\delta = 11\nu/u_*$ is the thickness of the viscous layer ($\delta = 0.15$ m in Heiskanen et al. (2014) (*50*)), $\kappa = 0.41$ is the von Kármán constant, $\nu = 1.35 \times 10^{-6}$ m^2 s^{-1} is the water kinematic viscosity and Sc_{CH_4} is the temperature-dependent Schmidt number of methane (*52*). The coefficients are $c_1 = 0.29, c_2 = 1, c_3 = 1$ in Read et al. (2012) (*49*), $c_1 = 0.5, c_2 = 0.77, c_3 = 0.3$ in Heiskanen et al. (2014) (*50*) and $c_1 = 0.4, c_2 = 0.77, c_3 = 0.56$ in Tedford et al. (2014) (*51*). The piston velocity [m s^{-1}] from MacIntyre et al. (2010) (*53*) was calculated as $k_{CH_4} = k_{600}(Sc_{CH_4}/600)^{-2/3}$ with $k_{600} = (2.04U_{w,10} + 2)/(3.6 \times 10^5)$ if $B^* > 0$ (cooling) or $k_{600} = (1.74U_{w,10} - 0.15)/(3.6 \times 10^5)$ if $B^* \leq 0$ (heating).

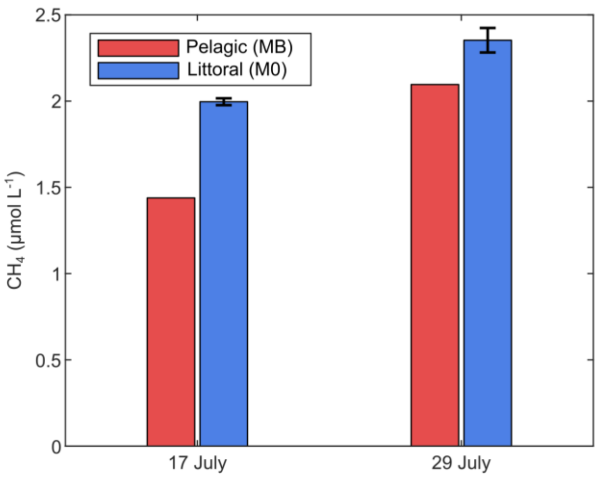

Fig. S8. Littoral and pelagic surface methane concentrations in summer (July 2019). Surface (1-2 m depth) methane concentrations were measured from water samples with the headspace method *(18)*. The error bars indicate the standard deviation between replicates. Littoral methane concentrations were constantly higher than pelagic concentrations, leading to a negative lateral methane gradient of $\partial CH_4/\partial x \approx -10^{-1}$ µmol L^{-1} km^{-1} between M0 and MB.

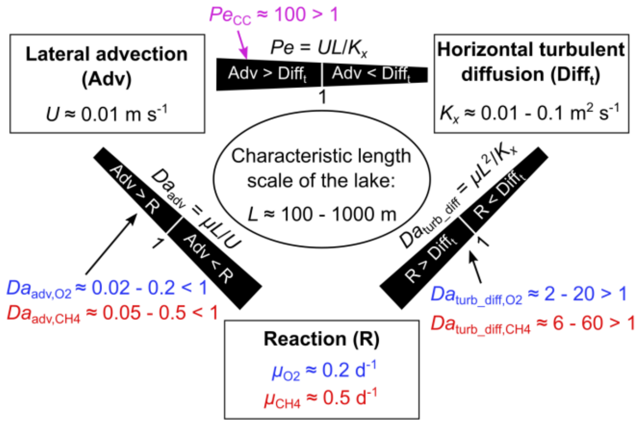

Fig. S9. Scaling approach assessing the contribution of lateral advection, horizontal turbulent diffusion and reaction to the mass balance of a dissolved substance. Each of the three dimensionless numbers compares the importance of two processes: the Péclet number *Pe*, advective Damköhler number Da_{adv} and dispersive Damköhler number Da_{disp} relate advection to turbulent diffusion, reaction to advection and reaction to turbulent diffusion, respectively. Parameter values specific to this study are shown (see supplementary text), where the characteristic length scale is between 100 and 1000 m, lateral advection is driven by convective circulation (CC) and dissolved constituents are oxygen (blue) and methane (red). In this example, lateral advection dominates the mass balance by exceeding fluxes associated with horizontal turbulent diffusion and reactions. In contrast, horizontal turbulent diffusion is not fast enough to overcome reaction rates.

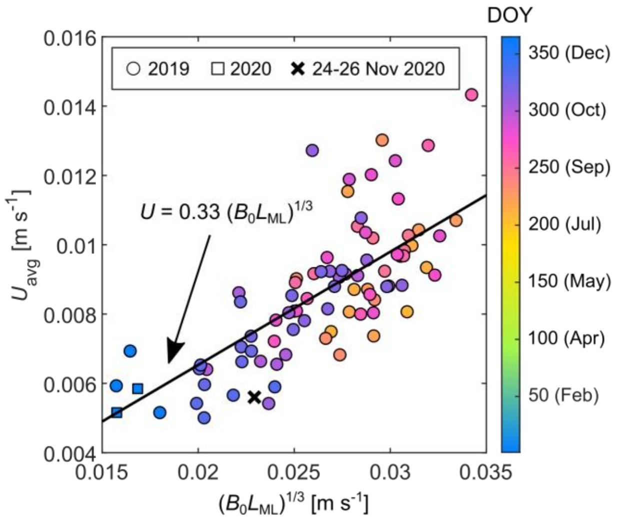

Fig. S10. Cross-shore velocity scaling of convective circulation. Daily averaged cross-shore velocity as a function of the velocity scale for 85 days with convective circulation between July 2019 and January 2020 (colored dots) and during the gas experiment (black cross). The black line is the linear fit $U = 0.33(B_0 L_{ML})^{1/3}$, where B_0 is the non-penetrative surface buoyancy flux (see fig. S2) and L_{ML} is the width of the mixed region shallower than the mixed layer depth, both averaged during the cooling period. This figure is the same as Fig. 6a in Doda et al. (2022) (*29*) with the additional data from the gas experiment.

Station	x (m)	Lake depth (m)	Location of the gas spectrometer	Measurement period (UTC)	Pump	Depth (m)	Height above sediment (m)	Sampling interval (min)
M0	0	1.6	Platform	24 Nov (16:15) – 26 Nov (11:00)	A	0.9	0.7	28
					B	1.3	0.3	28
M1	55	3.3	Boat	25 Nov (03:00-06:40)	E	0.4	2.9	14
					F	2.8	0.5	14
MT	93	4.2	Platform	24 Nov (16:15) – 26 Nov (11:00)	C	0.6	3.6	28
					D	3.8	0.4	28
M2	175	6.2	Boat	25 Nov (07:15-09:10)	E	0.4	5.8	14
					F	5.4	0.8	14
M3	259	8.7	Boat	25 Nov (22:15) – 26 Nov (11:00)	E	0.5	8.2	14
					F	7.6-8.3	0.4-1.1	14
MI	443	13.6	Boat	25 Nov (09:15-15:40)	E	9.7	3.9	14
					F	8.7-12.5	1.1-4.9	14
MB	792	15.9	Boat	25 Nov (16:45-21:20)	E	9.7	6.2	14
					F	0.5	15.4	14

Table S1. Characteristics of the measurement stations.

REFERENCES AND NOTES

1. R. de Groot, L. Brander, S. van der Ploeg, R. Costanza, F. Bernard, L. Braat, M. Christie, N. Crossman, A. Ghermandi, L. Hein, S. Hussain, P. Kumar, A. McVittie, R. Portela, L. C. Rodriguez, P. ten Brink, P. van Beukering, Global estimates of the value of ecosystems and their services in monetary units. *Ecosyst. Serv.* **1**, 50–61 (2012).

2. J.-P. Jenny, O. Anneville, F. Arnaud, Y. Baulaz, D. Bouffard, I. Domaizon, S. A. Bocaniov, N. Chèvre, M. Dittrich, J.-M. Dorioz, E. S. Dunlop, G. Dur, J. Guillard, T. Guinaldo, S. Jacquet, A. Jamoneau, Z. Jawed, E. Jeppesen, G. Krantzberg, J. Lenters, B. Leoni, M. Meybeck, V. Nava, T. Nõges, P. Nõges, M. Patelli, V. Pebbles, M.-E. Perga, S. Rasconi, C. R. Ruetz, L. Rudstam, N. Salmaso, S. Sapna, D. Straile, O. Tammeorg, M. R. Twiss, D. G. Uzarski, A.-M. Ventelä, W. F. Vincent, S. W. Wilhelm, S.-Å. Wängberg, G. A. Weyhenmeyer, Scientists' warning to humanity: Rapid degradation of the world's large lakes. *J. Great Lakes Res.* **46**, 686–702 (2020).

3. M. J. Vander Zanden, Y. Vadeboncoeur, Putting the lake back together 20 years later: What in the benthos have we learned about habitat linkages in lakes? *Inland Waters* **10**, 305–321 (2020).

4. K. Knauer, H. M. Nepf, H. F. Hemond, The production of chemical heterogeneity in Upper Mystic Lake. *Limnol. Oceanogr.* **45**, 1647–1654 (2000).

5. S. Brothers, G. Kazanjian, J. Köhler, U. Scharfenberger, S. Hilt, Convective mixing and high littoral production established systematic errors in the diel oxygen curves of a shallow, eutrophic lake. *Limnol Oceanogr-Meth* **15**, 429–435 (2017).

6. F. Peeters, H. Hofmann, Length-scale dependence of horizontal dispersion in the surface water of lakes. *Limnol. Oceanogr.* **60**, 1917–1934 (2015).

7. J. P. Antenucci, K. M. Tan, H. S. Eikaas, J. Imberger, The importance of transport processes and spatial gradients on in situ estimates of lake metabolism. *Hydrobiologia* **700**, 9–21 (2013).

8. S. Sadro, J. M. Melack, S. MacIntyre, Spatial and temporal variability in the ecosystem metabolism of a high-elevation lake: Integrating benthic and pelagic habitats. *Ecosystems* **14**, 1123–1140 (2011).

9. T. DelSontro, P. A. del Giorgio, Y. T. Prairie, No longer a paradox: The interaction between physical transport and biological processes explains the spatial distribution of surface water methane within and across lakes. *Ecosystems* **21**, 1073–1087 (2018).

10. C. Ordóñez, T. DelSontro, T. Langenegger, D. Donis, E. L. Suarez, D. F. McGinnis, Evaluation of the methane paradox in four adjacent pre-alpine lakes across a trophic gradient. *Nat. Commun.* **14**, 2165 (2023).

11. F. Peeters, J. Encinas Fernandez, H. Hofmann, Sediment fluxes rather than oxic methanogenesis explain diffusive CH_4 emissions from lakes and reservoirs. *Sci. Rep.* **9**, 243 (2019).

12. J. Murase, Y. Sakai, A. Sugimoto, K. Okubo, M. Sakamoto, Sources of dissolved methane in Lake Biwa. *Limnology* **4**, 91–99 (2003).

13. U. Tsunogai, Y. Miyoshi, T. Matsushita, D. D. Komatsu, M. Ito, C. Sukigara, F. Nakagawa, M. Maruo, Dual stable isotope characterization of excess methane in oxic waters of a mesotrophic lake. *Limnol. Oceanogr.* **65**, 2937–2952 (2020).

14. J. Encinas Fernández, F. Peeters, H. Hofmann, On the methane paradox: Transport from shallow water zones rather than in situ methanogenesis is the major source of CH_4 in the open surface water of lakes. *Eur. J. Vasc. Endovasc. Surg.* **121**, 2717–2726 (2016).

15. J. J. Coloso, J. J. Cole, P. C. Hanson, M. L. Pace, Depth-integrated, continuous estimates of metabolism in a clear-water lake. *Can. J. Fish. Aquat. Sci.* **65**, 712–722 (2008).

16. P. C. Hanson, S. R. Carpenter, N. Kimura, C. Wu, S. P. Cornelius, T. K. Kratz, Evaluation of metabolism models for free-water dissolved oxygen methods in lakes. *Limnol Oceanogr-Meth* **6**, 454–465 (2008).

17. P. A. Staehr, D. Bade, M. C. Van de Bogert, G. R. Koch, C. Williamson, P. Hanson, J. J. Cole, T. Kratz, Lake metabolism and the diel oxygen technique: State of the science. *Limnol Oceanogr-Meth* **8**, 628–644 (2010).

18. C. J. Schubert, F. S. Lucas, E. Durisch-Kaiser, R. Stierli, T. Diem, O. Scheidegger, F. Vazquez, B. Müller, Oxidation and emission of methane in a monomictic lake (Rotsee, Switzerland). *Aquat. Sci.* **72**, 455–466 (2010).

19. M. J. Czikowsky, S. MacIntyre, E. W. Tedford, J. Vidal, S. D. Miller, Effects of wind and buoyancy on carbon dioxide distribution and air-water flux of a stratified temperate lake. *Eur. J. Vasc. Endovasc. Surg.* **123**, 2305–2322 (2018).

20. H.-P. Grossart, K. Frindte, C. Dziallas, W. Eckert, K. W. Tang, Microbial methane production in oxygenated water column of an oligotrophic lake. *Proc. Natl. Acad. Sci. U.S.A.* **108**, 19657–19661 (2011).

21. M. Yao, C. Henny, J. A. Maresca, Freshwater bacteria release methane as a by-product of phosphorus acquisition. *Appl. Environ. Microbiol.* **82**, 6994–7003 (2016).

22. O. Mohseni, H. G. Stefan, D. Wright, G. J. Johnson, Dissolved oxygen depletion in a small deep lake with a large littoral zone. *Lake Reserv Manag.* **17**, 288–298 (2001).

23. B. J. Cardinale, T. M. Burton, V. J. Brady, The community dynamics of epiphytic midge larvae across the pelagic–littoral interface: Do animals respond to changes in the abiotic environment? *Can. J. Fish. Aquat. Sci.* **54**, 2314–2322 (1997).

24. L. C. Loken, J. T. Crawford, P. J. Schramm, P. Stadler, A. R. Desai, E. H. Stanley, Large spatial and temporal variability of carbon dioxide and methane in a eutrophic lake. *Eur. J. Vasc. Endovasc. Surg.* **124**, 2248–2266 (2019).

25. G. M. Horsch, H. G. Stefan, Convective circulation in littoral water due to surface cooling. *Limnol. Oceanogr.* **33**, 1068–1083 (1988).

26. S. G. Monismith, J. Imberger, M. L. Morison, Convective motions in the sidearm of a small reservoir. *Limnol. Oceanogr.* **35**, 1676–1702 (1990).

27. H. N. Ulloa, C. L. Ramón, T. Doda, A. Wüest, D. Bouffard, Development of overturning circulation in sloping waterbodies due to surface cooling. *J. Fluid Mech.* **930**, A18 (2022).

28. T. Doda, Density currents driven by differential cooling in lakes: Occurrence, dynamics and implications for littoral-pelagic exchange, thesis, EPFL, Ecole Polytechnique Fédérale de Lausanne, Lausanne, Switzerland (2022).

29. T. Doda, C. L. Ramón, H. N. Ulloa, A. Wüest, D. Bouffard, Seasonality of density currents induced by differential cooling. *Hydrol. Earth Syst. Sci.* **26**, 331–353 (2022).

30. W. F. James, J. W. Barko, Estimation of phosphorus exchange between littoral and pelagic zones during nighttime convective circulation. *Limnol. Oceanogr.* **36**, 179–187 (1991).

31. I. Fer, U. Lemmin, S. A. Thorpe, Winter cascading of cold water in Lake Geneva. *J. Geophys. Res.* **107**, 3060 (2002).

32. S. Ó. Pálmarsson, S. G. Schladow, Exchange flow in a shallow lake embayment. *Ecol. Appl.* **18**, A89–A106 (2008).

33. M. G. Wells, B. Sherman, Stratification produced by surface cooling in lakes with significant shallow regions. *Limnol. Oceanogr.* **46**, 1747–1759 (2001).

34. D. Donis, S. Flury, A. Stöckli, J. E. Spangenberg, D. Vachon, D. F. McGinnis, Full-scale evaluation of methane production under oxic conditions in a mesotrophic lake. *Nat. Commun.* **8**, 1661 (2017).

35. Y. R. Rao, D. J. Schwab, Transport and mixing between the coastal and offshore waters in the Great Lakes: A review. *J. Great Lakes Res.* **33**, 202–218 (2007).

36. C. L. Ramón, H. N. Ulloa, T. Doda, D. Bouffard, Flushing the lake littoral region: The interaction of differential cooling and mild winds. *Water Resour. Res.* **58**, e2021WR030943 (2022).

37. T. Doda, H. N. Ulloa, C. L. Ramón, A. Wüest, D. Bouffard, Penetrative convection modifies the dynamics of downslope gravity currents. *Geophys. Res. Lett.* **50**, e2022GL100633 (2023).

38. M. S. Brennwald, M. Schmidt, J. Oser, R. Kipfer, A portable and autonomous mass spectrometric system for on-site environmental gas analysis. *Environ. Sci. Technol.* **50**, 13455–13463 (2016).

39. M. S. Brennwald, M. Peel, T. Blanc, Y. Tomonaga, R. Kipfer, P. Brunner, D. Hunkeler, New experimental tools to use noble gases as artificial tracers for groundwater flow. *Front. Water* **4**, 925294 (2022).

40. C. J. Schubert, T. Diem, W. Eugster, Methane emissions from a small wind shielded lake determined by eddy covariance, flux chambers, anchored funnels, and boundary model calculations: A comparison. *Environ. Sci. Technol.* **46**, 4515–4522 (2012).

41. S. Xiao, L. Liu, W. Wang, A. Lorke, J. Woodhouse, H.-P. Grossart, A Fast-Response Automated Gas Equilibrator (FaRAGE) for continuous in situ measurement of CH₄ and CO₂ dissolved in water. *Hydrol. Earth Syst. Sci.* **24**, 3871–3880 (2020).

42. M. Günthel, D. Donis, G. Kirillin, D. Ionescu, M. Bizic, D. F. McGinnis, H.-P. Grossart, K. W. Tang, Contribution of oxic methane production to surface methane emission in lakes and its global importance. *Nat. Commun.* **10**, 5497 (2019).

43. M. J. Mayr, M. Zimmermann, J. Dey, A. Brand, B. Wehrli, H. Bürgmann, Growth and rapid succession of methanotrophs effectively limit methane release during lake overturn. *Commun. Biol.* **3**, 108 (2020).

44. G. H. Lauster, P. C. Hanson, T. K. Kratz, Gross primary production and respiration differences among littoral and pelagic habitats in northern Wisconsin lakes. *Can. J. Fish. Aquat. Sci.* **63**, 1130–1141 (2006).

45. M. Zimmermann, M. J. Mayr, H. Bürgmann, W. Eugster, T. Steinsberger, B. Wehrli, A. Brand, D. Bouffard, Microbial methane oxidation efficiency and robustness during lake overturn. *Limnol. Oceanogr. Lett.* **6**, 320–328 (2021).

46. A. Brand, H. Bruderer, K. Oswald, C. Guggenheim, C. J. Schubert, B. Wehrli, Oxygenic primary production below the oxycline and its importance for redox dynamics. *Aquat. Sci.* **78**, 727–741 (2016).

47. G. Fink, M. Schmid, B. Wahl, T. Wolf, A. Wüest, Heat flux modifications related to climate-induced warming of large European lakes. *Water Resour. Res.* **50**, 2072–2085 (2014).

48. J. Imberger, The diurnal mixed layer1. *Limnol. Oceanogr.* **30**, 737–770 (1985).

49. J. S. Read, D. P. Hamilton, A. R. Desai, K. C. Rose, S. MacIntyre, J. D. Lenters, R. L. Smyth, P. C. Hanson, J. J. Cole, P. A. Staehr, J. A. Rusak, D. C. Pierson, J. D. Brookes, A. Laas, C. H. Wu, Lake-size dependency of wind shear and convection as controls on gas exchange. *Geophys. Res. Lett.* **39**, L09405 (2012).

50. J. J. Heiskanen, I. Mammarella, S. Haapanala, J. Pumpanen, T. Vesala, S. Macintyre, A. Ojala, Effects of cooling and internal wave motions on gas transfer coefficients in a boreal lake. *Tellus B Chem. Phys. Meteorol.* **66**, 22827 (2022).

51. E. W. Tedford, S. MacIntyre, S. D. Miller, M. J. Czikowsky, Similarity scaling of turbulence in a temperate lake during fall cooling. *J. Geophys. Res. Oceans* **119**, 4689–4713 (2014).

52. R. Wanninkhof, Relationship between wind speed and gas exchange over the ocean revisited: Gas exchange and wind speed over the ocean. *Limnol Oceanogr-Meth* **12**, 351–362 (2014).

53. S. MacIntyre, A. Jonsson, M. Jansson, J. Aberg, D. E. Turney, S. D. Miller, Buoyancy flux, turbulence, and the gas transfer coefficient in a stratified lake. *Geophys. Res. Lett.* **37**, L24604 (2010).
